# A systematic review of combination treatment strategies for osteoporosis

**DOI:** 10.1093/jbmrpl/ziaf165

**Published:** 2025-10-22

**Authors:** Smita Nayak, Susan L Greenspan

**Affiliations:** Berkeley Madonna, Inc., Albany, CA, United States; University of Pittsburgh School of Medicine, Pittsburgh, PA, United States

**Keywords:** osteoporosis, antiresorptives, anabolics, BMD, fracture prevention, systematic review

## Abstract

The evidence and scientific guidance regarding the efficacy of combination treatment strategies for osteoporosis is currently limited. We performed a systematic review of primary studies that evaluated the effect of combination bone-active medication therapy on BMD and/or fracture outcomes. Eleven studies that assessed 7 different combinations of medications were included. Evidence for superior efficacy of combination treatment compared with monotherapy was strong for only 3 medication combinations: teriparatide and denosumab, teriparatide and zoledronic acid, and alendronate and raloxifene. Three small studies that evaluated combination teriparatide and denosumab found evidence of statistically significant benefit for increasing BMD at the lumbar spine and/or hip more than either medication alone. One large study that evaluated combination teriparatide and zoledronic acid found that after 1 yr of treatment BMD at the lumbar spine increased significantly more in the combination treatment group than the zoledronic acid alone group, and BMD at the hip increased significantly more in the combination treatment group than the teriparatide alone group. This study also found significantly reduced risk of clinical fractures with combination therapy compared with zoledronic acid alone, but not when compared with teriparatide alone. Two studies that evaluated combination alendronate and raloxifene found evidence of significant benefit on BMD outcomes. We found insufficient evidence for superior efficacy of other bone-active medication combinations compared with monotherapy on either BMD outcomes or the very limited data on fracture outcomes. In conclusion, there is evidence supporting the use of combination treatment with teriparatide and denosumab, teriparatide and zoledronic acid, and alendronate and raloxifene for osteoporosis for increasing BMD more than monotherapy, although several of the studies showing benefit of these combinations are small, and data are lacking on fracture outcomes. These combinations can be considered for treatment of patients with osteoporosis; however, further larger studies would be useful to evaluate fracture outcomes as well as other combinations for potential efficacy.

## Introduction

Osteoporosis is a common bone disease that affects primarily older adults and increases risk of fractures, which have significant associated morbidity, mortality, and costs.^1–5^ There are several medications available to treat osteoporosis which increase bone mineral density (BMD) and reduce risk of fracture, including anabolic agents that build bone, such as teriparatide, romosozumab, and abaloparatide, and antiresorptive agents which primarily inhibit bone breakdown, including bisphosphonates (alendronate, ibandronate, risedronate, and zoledronic acid), denosumab, and raloxifene. These medications are most commonly used as monotherapy (one medication at a time), although there has been growing interest in potential use of multiple medications simultaneously, particularly for high risk patients with very low BMD or recent fractures, to increase bone density and reduce fracture risk to possibly a greater extent than with a single medication.^6^ However, there is limited evidence and scientific guidance regarding the efficacy of combination treatment strategies, and this topic has been identified by experts, professional societies, and the NIH as an important area for future investigation.^6–8^ We performed a systematic review of primary studies that evaluated the effect of combination bone-active medication strategies on BMD and fracture outcomes.

## Materials and methods

### Data sources and search strategies

We performed literature searches in Embase, PubMed, and Cochrane Library databases in March 2025 to find primary studies evaluating the efficacy of combination medication treatment for osteoporosis; Table S1 shows the PubMed search strategy. The search strategies were designed to be broad to have high sensitivity for identifying potentially relevant studies and were limited to studies published in English in the year 2000 or later. Several additional studies were found by reviewing the reference lists of studies identified with the database searches.

### Study selection

Inclusion and exclusion criteria were applied to the literature found with the search strategies to identify relevant studies for this systematic review. Studies were required to evaluate combination therapy with at least two FDA-approved medications for treatment of osteoporosis compared to a comparator treatment (eg, a single medication) or placebo; be a clinical trial or cohort study; be published in English; require study participants to have osteoporosis or BMD T-scores <−2.0 at the spine or hip or spine, prior fragility fracture, or meet FRAX treatment criteria; report outcomes of either BMD at the hip or spine or fractures; have a duration of at least 1 yr; use doses of medication that are currently available in clinical practice; and report original results. We excluded studies that did not meet all of these criteria. The literature was evaluated for inclusion in two stages; titles and abstracts were screened first, and potentially relevant studies then received independent full-text review by the two study authors.

### Data extraction

Information extracted from included studies comprised location, publication year, participant demographic characteristics, study design and duration, medication combination evaluated, comparator treatment(s) evaluated, BMD outcomes, fracture outcomes, serious adverse events reported, and whether authors had received any pharmaceutical company funds.

### Data analysis

Our initial strategy was to perform meta-analyses for the efficacy of different combination treatment strategies on BMD outcomes, but the included studies were too heterogeneous to combine; specifically, the studies which evaluated the same combination strategies reported outcomes for different treatment durations. Thus, we instead described the findings of this systematic review qualitatively. We also assessed the methodological quality of included studies using Cochrane Collaboration risk of bias criteria.^9^

## Results

### Literature search and study selection

**Figure 1:**
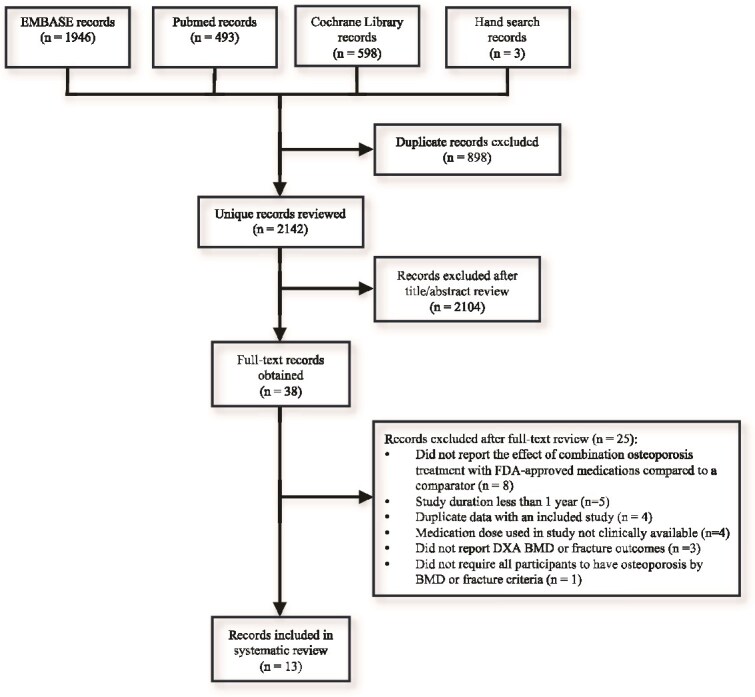
Flow diagram of literature search and study selection.

We identified 3040 records for review with the literature search strategies; after duplicate records identified by separate databases were excluded, there were 2142 unique records. Application of inclusion/exclusion criteria to these records identified 13 articles for inclusion,^10–22^ as shown in the flow diagram (Figure 1).

### Study characteristics

Table 1 shows the characteristics of included studies. The 13 included articles were published between 2002 and 2019 and presented results from 11 separate studies, 10 of which were randomized controlled trials. Four studies were performed in North America (all in the United States), 4 in Asia, 1 in Europe, and 2 on multiple continents. Number of study participants varied from 16 to 412; 8 studies included only postmenopausal women, 2 included women and men, and 1 included only men. Study duration varied from 1 to 4 yr. Seven different bone-active medication combinations were evaluated: teriparatide combined with denosumab was evaluated by 4 studies, teriparatide and alendronate by 2 studies, alendronate and raloxifene by 2 studies, and teriparatide and zoledronic acid, teriparatide and raloxifene, teriparatide and risedronate, and teriparatide and ibandronate by 1 study each. BMD outcomes at the lumbar spine (LS), total hip (TH), and/or femoral neck (FN) were reported by all included studies, but only 5 of the studies reported fracture outcomes, one of which captured fractures only as adverse events,^10^ although no study was statistically powered for fracture outcomes. No study reported a greater incidence of serious adverse events in the combination therapy group than the comparator group. Approximately two-thirds of the studies had authors who disclosed receiving pharmaceutical company funds.

**Table 1:** Included study characteristics and outcomes.

**First author, year, country**	**Study participants and description**	**Combination treatment evaluated**	**Comparator treatment**	**Number of participants included in analysis**	**Relevant outcome results**
**Cosman, 2011,** ^**10**^ **multiple countries**	Postmenopausal women age 45-89 yr with osteoporosis without prior long-term use of bisphosphonates or PTH; randomized partial double-blinded trial	Zoledronic acid 5 mg infusion × 1 and teriparatide 20 mcg daily	1. Zoledronic acid 5 mg infusion × 1 2. Teriparatide 20 mcg daily and placebo infusion x 1	412 (137 zoledronic acid and teriparatide/137 zoledronic acid alone/138 teriparatide plus IV placebo)	BMD percent change: LS, 1 yr: zoledronic acid and teriparatide: 7.5% zoledronic acid: 4.4% teriparatide: 7.0% (*p* < .001 for combination therapy vs zoledronic acid) TH, 1 yr: zoledronic acid and teriparatide: 2.3% zoledronic acid: 2.2% teriparatide: 1.1% (*p* ≤ .02 for combination therapy vs teriparatide) FN, 1 yr: zoledronic acid and teriparatide: 2.2% zoledronic acid: 1.9%* teriparatide: 0.1% (*p* < .001 for combination therapy vs teriparatide) Clinical fractures: zoledronic acid and teriparatide: 4 out of 137 (2.9%) zoledronic acid: 13 out of 137 (9.5%) teriparatide: 8 out of 137 (5.8%) (risk ratio for clinical fracture incidence for combination group compared to zoledronic acid group was 0.31; 95% CI, 0.10-0.92; *p* = .04; there was no significant difference in clinical fracture incidence for combination therapy group compared to teriparatide group) Serious adverse events: No significant differences in serious adverse events among groups
**Cosman, 2015,** ^**11**^ **USA**	Postmenopausal women over age 45 yr with osteoporosis; in the alendronate treatment arm of this study women had to have been taking alendronate for at least 1 yr; other arm was considered treatment naïve; randomized open-label study	1. Teriparatide 20 mcg daily and alendronate 70 mg weekly for 2 yr 2. Cyclic teriparatide (20 mcg daily for 3-mo cycles followed by 3 mo without teriparatide) and alendronate 70 mg weekly for 2 yr	1. Teriparatide 20 mcg daily for 2 yr 2. Cyclic teriparatide (20 mcg daily for 3-mo cycles followed by 3 mo without teriparatide) for 2 yr	150 (30 daily teriparatide and alendronate/34 cyclic teriparatide and alendronate/43 daily teriparatide/43 cyclic teriparatide	BMD percent change: LS, 2 yr: daily teriparatide and alendronate: 7.5% cyclic teriparatide and alendronate: 6.0% daily teriparatide: 8.8% cyclic teriparatide: 4.8% TH, 2 yr: daily teriparatide and alendronate: 3.0% cyclic teriparatide and alendronate: 2.5% daily teriparatide: 4% cyclic teriparatide: 2.1% FN, 2 yr: daily teriparatide and alendronate: 2.9% cyclic teriparatide and alendronate: 1.5% daily teriparatide: 2.9% cyclic teriparatide: 1.2% Fractures: Clinical fractures: daily teriparatide and alendronate: 3 out of 30 cyclic teriparatide and alendronate: 2 out of 34 daily teriparatide: 2 out of 43 cyclic teriparatide: 1 out of 43 New morphometric vertebral fractures: daily teriparatide and alendronate: 1 out of 30 cyclic teriparatide and alendronate: 1 out of 34 daily teriparatide: 1 out of 43 cyclic teriparatide: 1 out of 43 Serious adverse events: 34 serious adverse events occurred in 24 participants, balanced across groups, and apparently unrelated to teriparatide
**Cosman, 2009,** ^**12**^ **USA**	Postmenopausal women age 50 yr and older with osteoporosis on alendronate or raloxifene for at least 18 mo; randomized, open-label trial	1. Alendronate 70 total mg weekly for at least 18 mo followed by teriparatide 20 mcg daily + alendronate 70 mg weekly for 18 mo (alendronate add group) 2. Raloxifene 60 mg daily for at least 18 mo followed by teriparatide 20 mcg daily + raloxifene 60 mg daily for 18 mo (raloxifene add group)	1. Alendronate 70 total mg weekly for at least 18 mo followed by teriparatide 20 mcg daily for 18 mo (alendronate switch group) 2. Raloxifene 60 mg daily for at least 18 mo followed by teriparatide 20 mcg daily for 18 mo (raloxifene switch group)	167 (45 alendronate add /39 raloxifene add/45 alendronate switch/38 raloxifene switch)	BMD percent change: LS, before teriparatide initiation through month 18 of teriparatide treatment: alendronate add: 8.4% raloxifene add: 9.2% alendronate switch: 4.8% raloxifene switch: 8.1% (*p* = .003 for difference between alendronate add and alendronate switch; *p* not significant for difference between raloxifene add and raloxifene switch) TH, before teriparatide initiation through month 18 of teriparatide treatment: alendronate add: 3.2% raloxifene add: 2.8% alendronate switch: 0.9% raloxifene switch: 1.8% (*p* = .02 for difference between alendronate add and alendronate switch; *p* not significant for difference between raloxifene add and raloxifene switch) FN, before teriparatide initiation through month 18 of teriparatide treatment: alendronate add: 2.7% raloxifene add: 3.8% alendronate switch: 2.3% raloxifene switch: 2.2% (*p* not significant for difference between alendronate add and alendronate switch or difference between raloxifene add and raloxifene switch) Fracture outcomes: Clinical fractures: alendronate add: 6% (3 of 52 patients) raloxifene add: 6% (3 of 47 patients) alendronate switch: 10% (5 of 50 patients) raloxifene switch: 12% (6 of 49 patients) Serious adverse events: alendronate add: 6% raloxifene add: 11% alendronate switch: 16% raloxifene switch: 14% (*p* = .12 for comparison of alendronate add to alendronate switch, *p* = .76 for comparison of raloxifene add to raloxifene switch)
**Ide, 2018,** ^**13**^ **Japan**	Patients with osteoporosis and lumbar canal stenosis (mean age 74 ± 7 yr) undergoing surgery (posterior lumbar decompression and interbody fusion); RCT	Teriparatide 20 mcg daily (starting 1 mo before surgery and continuing for 1 yr after) and denosumab 60 mg every 6 mo × 2 at 2 and 8 mo after surgery	Teriparatide 20mcg daily (starting 1 mo before surgery and continuing for 1 yr after)	16 (8 teriparatide and denosumab/8 teriparatide only	BMD percent change: FN, 1 yr: teriparatide and denosumab: 2.4%* teriparatide: −4.0%* (*p* < .05) Serious adverse events: No adverse events in either group
**Idolazzi, 2016,** ^**14**^ **Italy**	Women over 65 yr old with severe postmenopausal osteoporosis (at least 2 moderate or severe vertebral fractures); open-label prospective study	Teriparatide 20 mcg daily (starting 3 mo after first denosumab administration) and denosumab 60 mg every 6 mo	1. Teriparatide 20 mcg daily 2. Denosumab 60 mg every 6 mo	59 (19 combination/20 teriparatide only/20 denosumab only)	BMD percent change: LS, 1 yr: teriparatide and denosumab: 7.3% ± 3.6 teriparatide: 6.5% ± 2.7 denosumab: 5.4% ± 3.0 No significant differences among groups with ANOVA analysis TH, 1 yr: teriparatide and denosumab: 3.8% ± 2.1 teriparatide: 0.0% ± 1.7 denosumab: 2.8% ± 1.9 No significant differences among groups with ANOVA analysis
**Johnell, 2002,** ^**15**^ **multiple countries**	Postmenopausal ambulatory women age ≤75 yr with low FN BMD; randomized, double-blind clinical trial	Raloxifene 60 mg daily and alendronate 10 mg daily	1. Raloxifene 60 mg daily 2. Alendronate 10 mg daily 3. Placebo	331 (84 raloxifene and alendronate/82 raloxifene only/83 alendronate only/82 placebo)	BMD percent change: LS, 1 yr: raloxifene and alendronate: 5.3% ± 0.4 raloxifene: 2.1% ± 0.4 alendronate: 4.3% ± 0.4 placebo: −0.004 ± 0.3 (*p*-value < .05 for comparison of combination treatment to raloxifene; *p* = .10 for comparison of combination treatment to alendronate) FN, 1 yr: raloxifene and alendronate: 3.7% ± 0.5 raloxifene: 1.7% ± 0.4 alendronate: 2.7% ± 0.5 placebo: 0.2% ± 0.4 (*p* < .001 for comparison of combination treatment to raloxifene; *p* = .02 for comparison of combination treatment to alendronate)
**Leder, 2014** ^**16**^ **and Tsai, 2013,**^**17**^ **USA**	Postmenopausal women age ≥45 yr with osteoporosis; open-label RCT	Teriparatide 20 mcg daily and denosumab 60 mg every 6 mo	1. Teriparatide 20 mcg daily 2. Denosumab 60 mg every 6 mo	94 (30 combination/31 teriparatide only/33 denosumab only)	BMD percent change: Posterior-anterior LS, 1 yr: teriparatide and denosumab: 9.1% (SD 3.9) teriparatide: 6.2% (SD 4.6) denosumab: 5.5% (SD 3.3) (*p* = .0139 for comparison of combination treatment to teriparatide; *p* = .0005 for comparison of combination treatment to denosumab) Posterior-anterior LS, 2 yr: teriparatide and denosumab: 12.9% ± 5.0 teriparatide: 9.5% ± 5.9 denosumab: 8.3% ± 3.4 (*p* = .01 for comparison of combination treatment to teriparatide; *p* = .008 for comparison of combination treatment to denosumab) TH, 1 yr: teriparatide and denosumab: 4.9% (SD 2.9) teriparatide: 0.7% (SD 2.7) denosumab: 2.5% (SD 2.6) (*p* < .0001 for comparison of combination treatment to teriparatide; *p* = .0011 for comparison of combination treatment to denosumab) TH, 2 yr: teriparatide and denosumab: 6.3% ± 2.6 teriparatide: 2.0% ± 3.0 denosumab: 3.2% ± 2.5 (*p* < .001 for comparisons of combination treatment to teriparatide or denosumab) FN, 1 yr: teriparatide and denosumab: 4.2% (SD 3.0) teriparatide: 0.8% (SD 4.1) denosumab: 2.1% (SD 3.8) (*p* = .0007 for comparison of combination treatment to teriparatide; *p* = .0238 for comparison of combination treatment to denosumab) FN, 2 yr: teriparatide and denosumab: 6.8% ± 3.6 teriparatide: 2.8% ± 3.9 denosumab: 4.1% ± 3.8 (*p* = .003 for comparison of combination treatment to teriparatide; *p* = .008 for comparison of combination treatment to denosumab) Serious adverse events: Year 1: 2 in teriparatide and denosumab group, 3 in teriparatide group, and 1 in denosumab group, all judged to be unrelated to study treatments. Year 2: 1 in teriparatide group and 1 in denosumab group, judged to be unrelated to study treatments.
**Nakamura, 2017** ^**18**^ **and Suzuki, 2019,**^**19**^ **Japan**	Japanese women with postmenopausal osteoporosis who were treatment-naïve; RCT	Teriparatide 20 mcg daily and denosumab 60 mg every 6 mo	Denosumab 60 mg every 6 mo	30 at 2 yr (17 teriparatide and denosumab/13 denosumab alone); 37 at 4 yr (17 teriparatide and denosumab/20 denosumab alone)	BMD percent change: LS, 2 yr: teriparatide and denosumab: 17.2% denosumab: 9.6% (*p* < .05) LS, 4 yr: teriparatide and denosumab: 21.1% denosumab: 14.2% No significant difference between the groups TH, 2 yr: teriparatide and denosumab: 9.5% denosumab: 5.6% No significant difference between the groups TH, 4 yr: teriparatide and denosumab: 9.7% denosumab: 6.8% No significant difference between the groups Fractures: No fractures reported in either group during 4-yr study Serious adverse events: No serious adverse events observed during 4-yr study
**Um, 2017,** ^**20**^ **South Korea**	Postmenopausal ambulatory women age ≤ 75 with established osteoporosis; RCT	Raloxifene 60 mg daily, alendronate 5 mg daily, and calcitriol 0.5 mcg daily	1. Raloxifene 60 mg daily 2. Alendronate 5 mg daily and calcitriol 0.5 mg daily 3. Control (1.0 g elemental calcium and 400 units of vitamin D daily); all study participants including those in treatment groups received calcium and vitamin D daily	62 (15 raloxifene, alendronate, and calcitriol/16 raloxifene only/17 alendronate and calcitriol only/14 placebo)	BMD percent change: LS, 3 yr: raloxifene and alendronate: 7.2% ± 0.5 raloxifene: 4.36% ± 0.3 alendronate: 6.7% ± 0.5 control: −1.81 ± 0.2 (*p* < .01 for comparison of combination treatment to raloxifene or alendronate) FN, 3 yr: raloxifene and alendronate: 4.8% ± 0.4 raloxifene: 1.9% ± 0.5 alendronate: 3.1% ± 0.3 control: −1.6% ± 0.5 (*p* < .01 for comparison of combination treatment to raloxifene or alendronate) Serious adverse events: No serious adverse events reported
**Walker, 2013,** ^**21**^ **USA**	Men with age 30-85 yr with low BMD; randomized, double-blind study	Teriparatide 20 mcg daily and risedronate 35 mg weekly	1. Teriparatide 20 mcg daily plus placebo tablet 2. Risedronate 35 mg weekly plus placebo injection	29 (10 teriparatide and risedronate/9 teriparatide and placebo/10 risedronate and placebo)	BMD percent change: LS, 1 yr: teriparatide and risedronate: 2.82% ± 1.5 teriparatide: 7.21% ± 1.5 risedronate: 1.93% ± 1.5 (*p* = .04 for comparison of combination treatment to teriparatide; no significant difference between combination therapy and risedronate) LS, 18 mo: teriparatide and risedronate: 6.95% ± 1.9 teriparatide: 5.68% ± 1.9 risedronate: 3.76% ± 1.8 No significant between-group differences TH, 18 mo: teriparatide and risedronate: 3.86% ± 1.1 teriparatide: 0.29% ± 0.95 risedronate: 0.82% ± 0.95 (*p* = .02 for comparison of combination treatment to teriparatide; *p* = .04 for comparison of combination treatment to risedronate) FN, 18 mo: teriparatide and risedronate: 8.45% ± 1.8 teriparatide: 3.89% ± 1.7 risedronate: 0.50% ± 1.7 (*p* = .07 for comparison of combination treatment to teriparatide; *p* = .002 for comparison of combination treatment to risedronate) Fractures: Morphometric vertebral: teriparatide and risedronate: 1 teriparatide: 0 risedronate: 1 No significant between-group difference in new vertebral fractures (*p* = 1.00) Clinical: teriparatide and risedronate: 1 teriparatide: 0 risedronate: 1 (*p* = 1.00)
**Ziying, 2019,** ^**22**^ **China**	Elderly patients (age > 60 yr, 79% female) with lumbar or hip joint injury caused by osteoporosis; RCT	Teriparatide 20 mcg daily and ibandronate 2 mg × 1, then 3 mg every 3 mo	1. Ibandronate 2 mg × 1, then 3 mg every 3 mo	98 (49 teriparatide and ibandronate/49 ibandronate alone)	LS BMD (g/cm^2^): Prior to treatment initiation: teriparatide and ibandronate: 0.664 ± 0.024 ibandronate: 0.665 ± 0.021 After 12 mo of treatment: teriparatide and ibandronate: 0.729 ± 0.028 ibandronate: 0.671 ± 0.021 (*p* < .001) FN BMD (g/cm^2^): Prior to treatment initiation: teriparatide and ibandronate: 0.532 ± 0.021 ibandronate: 0.535 ± 0.019 After 12 mo of treatment: teriparatide and ibandronate: 0.578 ± 0.025 ibandronate: 0.560 ± 0.028 (*p* < .001) Serious adverse events: There were no serious adverse events in either group

a Estimated from graph presented in article.

Abbreviation: RCT, randomized controlled trial.

### Assessment of potential sources of bias

Table S2 shows findings of the assessment of potential sources of bias/study quality. Of the 11 included studies, 9 were assessed to have high risk of bias and 2 were assessed to have an unclear risk of bias. All of the studies that were assessed as having high risk of bias were due to lack of blinding of participants and personnel; most studies were open-label in design, which resulted in high-risk assessment using the Cochrane criteria.^9^ However, despite this study design limitation, we believe the potential for bias in these studies was mitigated by the clinical outcomes of interest (BMD and fracture) being objective measurements. Additionally, 5 of these studies reported that the outcomes were assessed by individuals unaware of treatment assignment, which also reduced their susceptibility to bias. With respect to the studies that received an overall assessment of unclear risk of bias, both were judged as potentially having selection bias due to lack of information provided regarding allocation concealment; however, we believe that the potential impact of this was also reduced by the outcomes being objective measurements. All studies were assessed as having low risk of attrition bias due to incomplete outcome data or reporting bias due to selective reporting.

### Summary of findings for combination treatment strategies

#### Teriparatide and denosumab

Leder et al. and Tsai et al. compared teriparatide 20 mcg daily and denosumab 60 mg every 6 mo to teriparatide or denosumab alone in 94 postmenopausal women with osteoporosis, and found that combination treatment increased BMD at the spine and hip more than either medication alone 1 and 2 yr after treatment initiation.^16^^,^^17^ At 2 yr, mean percentage change in LS/TH/FN BMD was 12.9/6.3/6.8 in the combination teriparatide and denosumab group, 9.5/2.0/2.8 in the teriparatide only group, and 8.3/3.2/4.1in the denosumab only group; *p* values for comparisons of combination treatment to teriparatide or denosumab were less than or equal to .01 for all sites.^16^ Idolazzi et al. compared teriparatide 20 mcg daily (starting 3 mo after first denosumab dose) and denosumab 60 mg every 6 mo to teriparatide or denosumab alone in 59 women over 65 yr old with postmenopausal osteoporosis who were not on current therapy with any drug affecting the skeleton, and found no significant difference between the groups in mean BMD change at the LS or TH at 1 yr after denosumab initiation (9 mo after initiation of combination treatment).^14^ The lack of a significant finding for combination therapy in this study may have been affected by the 3 mo shorter treatment period of combination therapy (only 9 mo of combination therapy) compared to either of the monotherapy arms.^14^ Nakamura et al. evaluated teriparatide 20 mcg daily and denosumab 60 mg every 6 mo compared with denosumab alone in 30 women with postmenopausal osteoporosis who were treatment-naïve, and found that after 2 yr of therapy, mean BMD change at the LS was significantly greater in the teriparatide and denosumab group (17.2%) than the denosumab alone group (9.6%), *p* < .05.^18^ This study did not find a significant difference in mean BMD change at the TH, although the mean BMD change was 9.5% in the combination treatment groups vs 5.6% in the denosumab alone group (*p* value not provided).^18^ A paper by Suzuki et al. from this same study also provided BMD outcomes after 4 yr of treatment for 37 women; although mean BMD change at the LS and TH was greater with combination therapy than denosumab alone (LS, 21.1% vs 14.2%, respectively; TH, 9.7% vs 6.8%, respectively), these differences were not statistically significant (*p* values not provided); however, this study’s small size likely limited its power to detect a significant difference between treatment groups.^19^ Ide and colleagues compared teriparatide 20 mcg daily and denosumab 60 mg every 6 mo to teriparatide alone in 16 participants with osteoporosis and lumbar canal stenosis undergoing surgery, and reported that combination treatment increased FN BMD more than teriparatide alone at 1 yr (estimated 2.4% vs −4.0%, respectively, *p* < .05).^13^

#### Teriparatide and bisphosphonates

Cosman et al. evaluated combination teriparatide 20 mcg daily and zoledronic acid 5 mg one-time infusion to either drug alone in 412 postmenopausal women with osteoporosis who were treatment-naïve, and found that at 1 yr LS BMD increased more in the combination treatment group than zoledronic acid alone group (7.5% vs 4.4%, *p* < .001), but no significant difference compared with teriparatide alone (7.0%).^10^ BMD at the TH and FN increased more at 1 yr in the combination treatment group than teriparatide alone group (2.3% and 2.2% vs 1.1% and 0.1%, respectively, *p* values ≤ .02 and <.001, respectively), but there was no significant difference with combination treatment compared with zoledronic acid alone at these sites.^10^ This study also found that there were significantly fewer incident clinical fractures in the combination treatment group than the zoledronic acid group; 2.9% of the combination therapy group experienced fractures vs 9.5% of the zoledronic acid group, for a risk ratio of 0.31 (95% CI, 0.10-0.92, *p* = .04).^10^ There was no significant difference in incident clinical fractures in the teriparatide group compared with the combination therapy group.^10^

Teriparatide and alendronate were evaluated in 2 studies in which participants were postmenopausal women who had already taken alendronate for at least 1 yr.^11^^,^^12^ Cosman et al. compared teriparatide 20 mcg daily and alendronate 70 mg weekly to teriparatide alone for 18 mo in 90 postmenopausal women with osteoporosis who had already taken alendronate for at least 18 mo, and found significantly greater mean BMD gain in the combination treatment group at the LS (8.4% vs 4.8%, *p* = .003) and TH (3.2% vs 0.9%, *p* = .02), but not at the FN (2.7% vs 2.3%, *p* = .75).^12^ In another study with 150 participants, Cosman et al. compared either daily or 3-mo cyclic therapy with teriparatide 20 mcg daily combined with alendronate 70 mg weekly in postmenopausal women with osteoporosis who had already taken alendronate for at least 1 yr to daily or cyclic teriparatide alone in treatment-naïve women, and found similar BMD increases in the combination treatment groups compared to the teriparatide-alone groups after 2 yr of treatment.^11^

A small study by Walker et al. with 29 male participants with low BMD who had not used any osteoporosis therapy within the prior 6 mo evaluated teriparatide 20 mcg daily and risedronate 35 mg weekly compared to either drug alone for 18 mo, and found greater increase in BMD at the TH but not FN or LS with combination treatment than monotherapy with either drug; at the TH BMD increase was 3.86% with combination therapy vs 0.29% with teriparatide alone and 0.82% with risedronate alone (*p* = .02 for comparison of combination treatment to teriparatide alone, and *p* = .04 for comparison of combination treatment to risedronate alone), and at the FN BMD increase was 8.45% with combination therapy vs 3.89% with teriparatide alone and 0.50% with risedronate alone (*p* = .07 for comparison of combination treatment to teriparatide alone, and *p* = .002 for comparison of combination treatment to risedronate alone).^21^ A study by Ziying and Ping with 98 older participants with osteoporosis who had not recently taken osteoporosis therapy compared combination teriparatide and ibandronate treatment for 1 yr to ibandronate alone, and found significantly greater BMD at the LS and FN with combination treatment than ibandronate alone (*p* values for both comparisons <.001, BMD percent change not provided); however, this study did not include a teriparatide alone group.^22^

#### Alendronate and raloxifene

Johnell et al. evaluated 1 yr of treatment with alendronate 10 mg daily combined with raloxifene 60 mg daily to either medication alone or placebo in 331 postmenopausal ambulatory women age ≤75 yr, and found that at the LS combination therapy resulted in significantly larger increases in BMD than the raloxifene only group (5.3% vs 2.1%, *p* < .05), but no significant difference when compared with the alendronate only group (4.3%, *p* = .10).^15^ At the FN, the investigators found that combination alendronate and raloxifene increased BMD significantly more than either mediation alone, with a 3.7% increase in the combination therapy group compared to 2.7% in the alendronate only group and 1.7% in the raloxifene only group (*p* = .02 for comparison of combination treatment to alendronate alone, and *p* < .001 for comparison of combination treatment to raloxifene alone).^15^ A study by Um et al. that evaluated 3 yr of combination treatment with alendronate 5 mg daily and raloxifene 60 mg daily to either medication alone or placebo in 62 postmenopausal women with osteoporosis age 75 yr and younger found that combination therapy resulted in significantly larger mean increases in BMD at the LS and FN (7.2% and 4.8%, respectively) than alendronate alone (6.7% and 3.1%, respectively) or raloxifene alone (4.36% and 1.9%, respectively), all *p* values <.01.^20^

#### Teriparatide and raloxifene

Cosman et al. compared 18 mo of treatment with teriparatide 20 mcg daily and raloxifene 60 mg daily to teriparatide alone in 77 postmenopausal women with osteoporosis who had already been taking raloxifene for at least 18 mo, and found that although BMD increases were numerically greater in the combination therapy group than the teriparatide alone group at the LS (9.2% vs 8.1%), TH (2.8% vs 1.8%), and FN (3.8% vs 2.2%) at 18 mo, these differences were not significant.^12^

## Discussion

This systematic review of combination medication treatment strategies for osteoporosis identified 11 primary studies that assessed 7 different combinations of bone-active medications. All included studies reported BMD outcomes, however most did not report fracture outcomes. We found limited evidence of superior efficacy for BMD outcomes of combination treatment compared with monotherapy, with strong evidence for only 3 medication combinations: teriparatide and denosumab, teriparatide and zoledronic acid, and alendronate and raloxifene. The findings of this systematic review are summarized below.

### Combinations with evidence of benefit

Three small studies that evaluated combination teriparatide 20 mcg daily and denosumab 60 mg every 6 mo found evidence of stasticially significant benefit for increasing BMD at the LS and/or hip more than either teriparatide or denosumab alone.^13^^,^^16–18^ The largest of these studies included 94 postmenopausal women with osteoporosis and found that combination teriparatide and denosumab was superior to either medication alone for increasing LS, TH, and FN BMD at 1 and 2 yr after treatment initiation.^16^^,^^17^ In the single large study which included 412 postmenopausal women with osteoporosis that evaluated combination teriparatide 20 mcg daily and zoledronic acid 5 mg one-time infusion compared with either medication alone, combination therapy was found to be superior to either medication alone when considering both LS and hip BMD outcomes after 1 yr of treatment.^10^ This study also found significant benefit of combination teriparatide and zoledronic acid compared with zoledronic acid monotherapy with respect to clinical fracture outcomes.^10^ Both included studies that evaluated combination alendronate and raloxifene found significant benefit compared with monotherapy with either medication; the larger of these studies included 331 postmenopausal women with low BMD and found that combination treatment was superior to monotherapy with either medication for increasing FN BMD.^15^^,^^20^

### Combinations for which evidence is insufficient

We found insufficient evidence for whether the combination of teriparatide with oral bisphosphonates may be superior to either drug alone. Cosman et al. compared teriparatide 20 mcg daily and alendronate 70 mg weekly to teriparatide alone for 18 mo in 90 postmenopausal women with osteoporosis who had already taken alendronate for at least 18 mo, and found significantly greater mean BMD gain in the combination treatment group at the LS and TH.^12^ However, in another study that included 150 postmenopausal women with osteoporosis, Cosman et al. found similar BMD increases with 2 yr of combination teriparatide 20 mcg daily and alendronate 70 mg weekly in women who had already taken alendronate for at least 1 yr to teriparatide alone in treatment-naïve women.^11^ Other studies evaluated teriparatide with risedronate or teriparatide plus ibandronate; these studies found benefit of teriparatide and an oral bisphosphonate on BMD outcomes at the hip compared with the oral bisphosphonate alone;^21^^,^^22^ one of these studies also showed benefit of combination teriparatide and risedronate compared with teriparatide alone on BMD outcomes at the TH,^21^ and the other study found benefit of combination teriparatide with ibandronate compared with ibandronate alone on BMD outcomes at the LS.^22^ We also found insufficient evidence to support combination teriparatide and raloxifene treatment.^12^

A prior systematic review and meta-analysis on combination therapy for osteoporosis by Lou et al. published in 2019 had a narrower focus on studies that combined PTH analogs and antiresorptive agents, and reported that combination therapy with PTH analogs and antiresorptive agents, in general, significantly increased BMD at the LS and TH and reduced the risk of fractures compared to monotherapy; however, this analysis had critical limitations of grouping studies together without consideration of important differences.^23^ Specifically, studies evaluating 7 different antiresorptive agents (alendronate, risedronate, zoledronate, ibandronate, raloxifene, denosumab, and hormone replacement therapy (HRT)) were analyzed together without separate analysis of specific medication combinations; study durations of 6-36 mo were considered collectively without subgroup analysis by duration; and studies which evaluated HRT were included in the broad antiresorptive category, although HRT is not FDA-approved for treatment of osteoporosis in the U.S. (it is only approved for prevention, and use for treatment of osteoporosis is limited to relatively rare and specific circumstances).^23^ Our study had a broader focus, evaluated the evidence for efficacy of 7 different specific combinations of bone-active medications compared to monotherapy with particular agents, and did not group studies with widely varying treatment durations together, as we did not feel it was clinically appropriate to do so.

This systematic review had limitations. No included study was statistically powered for fracture outcomes, and thus this review was primarily focused on BMD outcomes; however, BMD change has been shown to be strongly associated with fracture risk reduction.^24^ Most of the included studies limited participation to postmenopausal women, and thus our findings are most relevant to that population. Many included studies were relatively small, and some treatment combinations were evaluated by only 1 small study; larger primary studies would be useful to further evaluate treatment combinations for which evidence is currently insufficient, such as teriparatide and oral bisphosphonates and teriparatide and raloxifene, and other combinations for which studies are currently lacking, such as romosozumab and denosumab, romosozumab and bisphosphonates, and bisphosphonates and denosumab. Larger studies could also have sufficient power to evaluate potential benefit of combination treatment compared to monotherapy on fracture outcomes. Furthermore, additional studies could evaluate how treatment with monotherapy before combination therapy is started compared with starting combination therapy for treatment-naïve patients may affect the subsequent BMD response to combination therapy. Moreover, although no included studies reported a greater incidence of serious adverse events in the combination therapy group than the comparator group, studies were likely not adequately powered to detect rare adverse events, and we cannot rule out the possibility that combination treatment may be associated with increased risk of adverse events. Whether combination therapy for osteoporosis is worthwhile when taking into account added medication expenses and a possible increased risk of adverse events is beyond the scope of this review.

In summary, this systematic review of combination medication treatment strategies for osteoporosis found limited evidence for superior efficacy of combination treatment compared with monotherapy on BMD outcomes, with strong evidence for only 3 medication combinations out of the 7 evaluated by primary studies: teriparatide 20 mcg daily and denosumab 60 mg every 6 mo for 1 or 2 yr, teriparatide 20 mcg daily for 1 yr and zoledronic acid 5 mg one-time infusion, and alendronate 10 mg daily combined with raloxifene 60 mg daily for 1-3 yr. Evidence is insufficient for superior efficacy of other bone-active medication combinations compared with monotherapy, and additional larger clinical studies would be useful to evaluate other combinations for potential efficacy.

## Supplementary Material

Supplemental_Table_S1_ziaf165

Supplemental_Table_S2_ziaf165_Revision

## Data Availability

The data underlying this article are available in the article and its online supplemental material.
