# Supplementary material for: A systematic review of combination treatment strategies for osteoporosis
Source: JBMR Plus. 2025 Oct 22;9(12):ziaf165. doi: 10.1093/jbmrpl/ziaf165 (PMC12599303; doi:10.1093/jbmrpl/ziaf165)
Supplement: Supplemental_Table_S1_ziaf165 [file supplemental_table_s1_ziaf165.docx]

**Supplemental Table S1. PubMed Search Strategy**

| **Database** | **Search Strategy**  **** |
| --- | --- |
| PubMed | (("low bmd" OR "low bone mineral density" OR "low bone mass" OR "bone fragility" OR "low bone density" OR osteoporo* OR fracture* OR FRAX) AND ((english[Filter]) AND (2000:2025[pdat]))) AND ((teriparatide OR denosumab OR risedronate OR "risedronic acid" OR alendronate OR "alendronic acid" OR zoledronate OR "zoledronic acid" OR ibandronate OR "ibandronic acid" OR raloxifene OR abaloparatide OR romosozumab) AND ((english[Filter]) AND (2000:2025[pdat]))) AND ((combin* OR concurrent* OR concomitant OR coadminist* OR added OR overlapping) AND ((english[Filter]) AND (2000:2025[pdat]))) AND (("randomized controlled trial" OR (randomized [tiab] AND controlled [tiab] AND trial [tiab]) OR "controlled clinical trial" OR (control* [tiab] AND clinical [tiab] AND trial [tiab]) OR cohort) AND ((english[Filter]) AND (2000:2025[pdat]))) |
