# Supplementary material for: A systematic review of combination treatment strategies for osteoporosis
Source: JBMR Plus. 2025 Oct 22;9(12):ziaf165. doi: 10.1093/jbmrpl/ziaf165 (PMC12599303; doi:10.1093/jbmrpl/ziaf165)
Supplement: Supplemental_Table_S2_ziaf165_Revision [file supplemental_table_s2_ziaf165_revision.docx]

**Supplemental Table S2. Study Quality Assessment^a^**

| **Study** | **Bias domain** | | | | | | **Summary assessment of risk of bias** |
| --- | --- | --- | --- | --- | --- | --- | --- |
|  | **Selection bias** | | **Performance bias** | **Detection bias** | **Attrition bias** | **Reporting bias** |  |
|  | **Random sequence generation** | **Allocation concealment** | **Blinding of participants and personnel** | **Blinding of outcome assessment** | **Incomplete outcome data** | **Selective reporting** |  |
| Cosman, 2011^(10)^ | Low risk | Unclear risk | High risk | Low risk | Low risk | Low risk | High risk |
| Cosman, 2015^(11)^ | Unclear risk | Unclear risk | High risk | Low risk | Low risk | Low risk | High risk |
| Cosman, 2009^(12)^ | Low risk | Low risk | High risk | Unclear risk | Low risk | Low risk | High risk |
| Ide, 2018^(13)^ | Unclear risk | Unclear risk | High risk | Unclear risk | Low risk | Low risk | High risk |
| Idolazzi, 2016^(14)^ | N/A^b^ | N/A^b^ | High risk | Unclear risk | Low risk | Low risk | High risk |
| Johnell, 2002^(15)^ | Low risk | Unclear risk | Low risk | Low risk | Low risk | Low risk | Unclear risk |
| Leder, 2014^(16)^ & Tsai, 2013^(17)^ | Low risk | Unclear risk | High risk | Low risk | Low risk | Low risk | High risk |
| Nakamura, 2017^(18)^ & Suzuki, 2019^(19)^ | Unclear risk | Unclear risk | High risk | Low risk | Low risk | Low risk | High risk |
| Um, 2017^(20)^ | Unclear risk | Unclear risk | High risk | Low risk | Low risk | Low risk | High risk |
| Walker, 2013^(21)^ | Low risk | Unclear risk | Low risk | Low risk | Low risk | Low risk | Unclear risk |
| Ziying, 2019^(22)^ | Unclear risk | Unclear risk | High risk | Unclear risk | Low risk | Low risk | High risk |

^a^Study methodological quality assessed using Cochrane Collaboration risk of bias criteria

^b^Not applicable – not a randomized study
